# Effects of ginger extract on smooth muscle activity of sheep reticulum and rumen

**Published:** 2013

**Authors:** Amin Mamaghani, Masoud Maham, Bahram Dalir-Naghadeh

**Affiliations:** *Department of Clinical Sciences, Faculty of Veterinary Medicine, Urmia University, Urmia, Iran.*

**Keywords:** Electromyography, Ginger extract, Prokinetic, Reticulorumen hypomotility

## Abstract

Reticulorumen hypomotility leads to the impaired physiologic functions of the digestive tract. Prokinetic action of ginger has been demonstrated in the laboratory animals and human. The aim of this study was to evaluate the effect of hydroalcoholic extract of ginger on contraction and motility of reticulum and rumen of ruminants. Collected samples of reticulum and rumen from eight sheep were investigated *in vitro*. The extract at the concentration of 0.1 and 1.0 mg L^-1^ had no effect on any preparations. Contraction of reticulum and rumen preparations was occurred at 10.0 and 100 mg L^-1^ concentrations (*p* < 0.05). Concentration of 1000 mg L^-1^ caused a relaxation in preparations contracted with 10.0 and 100 mg L^-1^. Likewise, the concentration of 1000 mg L^-1^ significantly (*p* < 0.05) inhibited ACh-induced contraction in both tissues. Six sheep were involved in electromyographic study. Administration of 40 mg kg^-1^ of the extract increased the overall frequency of contractions of the reticulum and rumen at the subsequent three days with the prominent increase at the second day (*p* < 0.05). Results of *in vitro* study indicated that hydroalcoholic extract of ginger contained spasmogenic and spasmolytic constituents. The results *in vivo* study represented evidences that the extract may have stimulant effect on reticulorumen motility in 40 mg kg^-1^ concentration.

## Introduction

Reticuloruminal motility supports the basic physiologic functions of the gastrointestinal tract of the ruminants and is needed for rumination to proceed.^1^ Reticulorumen hypo-motility is believed to play an important role in the etio-pathogenesis of gastrointestinal disorders in adult ruminants. Hypomotility or even complete stasis of reticulorumen has been associated with several conditions.^2^ The principle of treatment of reticulorumen hypomotility is restoring the normal motility in addition to the correction of the causative agent.^1^ Treatment of ruminants suspected of having reticular hypomotility is widely practiced, but few data are available on treatment efficacy. Prokinetic agents can stimulate, coordinate, and restore gastric, pyloric, and small intestinal motility.^3^ On the notion that neostigmine or metoclopramide have prokinetic effects in domestic monogastric animals, some practitioners have administered these medications to treat the ruminant reticular hypo-motility.^4^^,^^5^ However, the efficacy of these agents is unknown in ruminants.^2^

A relatively new topic in ruminant nutrition is the study of dietary additives with natural origin such as plant products capable of modulating and improving reticulo-ruminal physiological functions.^6^^-^^8^

Rhizome of *Zingiber officinale *Roscoe (family: *Zingib-eraceae*), commonly known as ginger, is a widely used food additive and spice and a phytomedicine used since the ancient times.^9^ Ginger has been used for medicinal purpose for the treatment of a number of diseases including those affecting the digestive tract.^10^ Ginger is universally reputed for its use in gastrointestinal disorders as a stomachic, laxative and prokinetic, and at the same time as an antidiarrheal, antidysenteric, antispasmodic and anti-colic aid.^9^ Several studies in animals and humans are indicative of prokinetic action of the ginger.^11^

In the present study we have investigated the effect of the hydro-alcoholic extract of this herbal medicine on the contractility of the preparations of reticulum and rumen of sheep. Furthermore, to evaluate the effect of the extract on motility of the reticulum and rumen of conscious sheep we studied myoelectrical activity of these organs smooth muscles as a method for assessment of their contractions.^12^

## Materials and Methods

**Extraction procedure. **The extract of ginger was prepared with the method described earlier .^9^ A total of 4.0 kg fresh ginger rhizomes were washed with water and then sliced to small pieces and then soaked in 6.0 L of 70% (v/v) aqueous ethanol and kept for a total of three days (at room temperature). After three days it was filtrated through a porous cloth and the filtrate collected, while the plant material was again soaked in 6.0 L of 70% aqueous ethanol for three days, twice. The combined filtrate was filtered through filter paper and evaporated to obtain a thick, brown extract with a yield of 4.1 % (w/w).

**Drug and chemicals.** Acetylcholine chloride (ACh) was obtained from Sigma Chemical Company, USA. Stock solution of ACh and dilutions of ginger extract were prepared fresh in distilled water on the day of the experiment. The concentrations of the ACh and ginger extract to give final bath concentrations were 10^-6^ M and 1×10^-1^ to 1×10^3^ mg L^-1^ (logarithmic increment), respectively.

The composition of the Tyrode’s Ringer solution was (mM): calcium chloride (1.8), magnesium chloride (1.0), potassium chloride (2.7), sodium bicarbonate (11.9), sodium chloride (137.0), sodium dihydrogen orthophosphate (0.4) and D-glucose (5.5). Salts for solutions were obtained from Merck, Darmstadt, Germany.

**Experimental specimens and tissue preparation.** Whole wall samples were taken from the dorsal rumen and reticulum of pasture-fed male sheep and lambs (aged six to nine months), killed at the Urmia abattoir. Tissue from different animals was collected immediately (usually 5-10 min) after slaughtering of the animals and placed in a Tyrode’s Ringer solution at 4 ˚C and was transported to the laboratory for immediate use. Tissues were washed gently with Tyrode’s Ringer solution. The whole pieces of tissue were placed in a petri dish filled with Tyrode’s Ringer solution at room temperature and the mucosa was carefully removed from the muscle layers. The intact muscle layers of the reticulum and rumen wall were cut into strips (3 mm × 20 mm) in a direction that allowed recording of longitudinal muscle activity. The remaining tissues were stored in Tyrode’s Ringer solution at 4 ˚C.

**Organ bath study. **Tissue preparations from reticulum and rumen of sheep were suspended in four individual organ baths containing 25 mL Tyrode’s Ringer solution each. The solution was constantly gassed with 95% oxygen and 5% carbon dioxide during the study and the temperature was maintained at 37 ˚C. The preparations were distally attached to a hook and proximally connected to isometric force transducers (TRI 202P, PanLab, Barcelona, Spain) coupled to bridge amplifier (ML224, AD Instruments, Castle Hill, Australia) and data acquisition PowerLab system (ML870, AD Instruments, Castle Hill, Australia) by use of commercially available software (Chart, Version 6, AD instruments, Sydney, Australia). Basal tension of 2.0 g was applied and during this time Tyrode’s Ringer solution was replaced every 15 min with fresh solution. The tissues kept undistributed for an equilibration period of 60 min, and then responses to ACh (1.0 μM) were repeated to test for viability and stabilizing the preparations. A dose–response curve was obtained with adding the increasing concentrations of ACh to the baths with 1 min intervals. The curve was used to determine the effect of ACh. Then, the submaximal (70% maximal) concentration was determined for each of the strips. The tissues, which produced three consistent, repeatable responses to sub-maximal concentration of acetylcholine, were used.

In this study the effects of ginger extract on strips of reticulum and rumen were examined in two series of experiments for each tissue. In first series of experiments the ginger extract was tested on resting baseline of smooth muscle strips in an accumulative manner at 5-minute intervals at 0.1, 1.0, 10.0, 100 and 1000 mg L^-1^. In the second series of experiments, the mentioned concentrations of extract were applied (in the same manner and interval time) to ACh-induced contractions to test the relaxant or stimulant effects of the extract.

The solvent (distilled water) controls (0 mg L^-1^ extract) were performed for each experiment before adding the treatment concentrations of the extract.

At the end of each trial, the organ baths were flushed twice and a further 50 min of activity was recorded and then ACh was added to test the viability of preparations.

The effects of the ginger extract on smooth muscle preparation were observed from the chart recordings. Baseline was defined as pre-treatment contraction value and was used for further comparative analysis. The effects of the cumulative concentrations of ginger extract on the basal tone (BT) of the contractions were noted. In both series of experiments, effect of the extract was assessed as the percent of the maximum effect produced by ACh (1.0 μM). Quantitative data were obtained by the aforementioned computer program Chart and PowerLab system.

**Electromyographic study.** Six Makouei wethers, aged 8-10 months and weighing between 26 to 30 kg were used in the study. Sheep were maintained in individual cages in an experimental room and fed with diet of 1200 g of dried chaffed alfalfa hay and 100 g of concentrate pellets once a day at 6:00 pm. Remaining food was removed at 8:00 AM and weighed to determine the amount consumed. Water was freely available except during the experiments.

The electromyographic activity used as an index of motility of the reticulorumen was based on the method of Ruckebusch.^12^ In each sheep, with local anesthesia and aseptic surgical procedures, a set of 3 insulated silver electrode wires (A-M Systems, Inc., Carlsborg, WA, USA) with bared ends was sutured into the smooth musculature of the reticulum and the cranial and caudal aspects of the dorsal sac of the rumen. The electrode wires were exteriorized from the abdominal wall and secured in a plug on the sheep’s back. Such preparations were maintained and tested for at least three weeks before experiments were initiated. All electrodes were connected to amplifier (ML135, AD Instruments, Castle Hill, Australia) and then to the PowerLab (ML870, AD Instruments, Castle Hill, Australia).

All experiments were performed at the same cages where sheep were kept. Animals were made to remain standing during experiments by suspending a sling beneath their brisket. Sheep had become habituated to these procedures prior to the start of the experiments. Each experiment began at 9:00 AM and continued 6 hr. The concentration of the extract (40 mg kg^-1^), duration of recordings (6 hr at subsequent three days after extract administration) and appropriate interval between experiments (15 days) were chosen on the basis of pilot experiments.

Each sheep received on separate days distilled water (control group) or ginger extract (treatment group) intraruminally in accordance with a crossover design. One day before administration of distilled water or extract (D_B_) the baseline electromyographic activity of reticulum and rumen recorded normally for 6 hr. In the next day (D_0_) each sheep of treatment group received 40 mg kg^-1^ of ginger extract in 100 mL of distilled water and each sheep of control group received only 100 mL distilled water at 9:00 AM and electromyographic activity of rumen and reticulum recorded for 6 hr. In following three days (D_1_, D_2_ and D_3_) recordings were done in each group from 9:00 AM to 3:00 PM without administration of extract or distilled water.

Data were saved on disc using Chart software (Version 6, AD instruments, Sydney, Australia). Contractions were assessed as real contractions based on reviewed papers.^13^^-^^16^ Ruckebusch has validated this EMG technique for motility of the reticulum and rumen,^12^ and that allowed the distinction of “A” and “B” sequences of contraction of the reticulum and rumen. “A” sequences consist of contraction of the reticulum, followed by the rumen, whereas “B” sequences of contraction are of the rumen alone.^17^ The frequency of “A” and “B” sequences of contraction of the reticulum and rumen and the frequency of reticular contractions associated with rumination were counted from chart recordings and data were analyzed. In two sheep, the contractions of reticulum could not be counted, thus the data were discarded.

**Statistical analysis. **For organ bath study, Friedman repeated measures ANOVA test on ranks was used which followed by the Dunnett’s test for paired wise comparison between each treatment and the solvent (0 mg L^-1^ extract). For all comparisons, a value of *p* < 0.05 was considered significant. Data are presented as median and interquartile range (25% and 75% quartiles). Statistical analyses were performed using SigmaStat for Windows (Version 3.5, Systat Software Inc., Chicago, IL, USA).

Data of electromyographic parameters were analyzed using a two-period, two-treatment (i.e., the extract and distilled water) crossover design, with three week washout intervals was used in a randomized sequence. The results were analyzed using a repeated measures ANOVA with Proc Mixed. Differences between least squares means were determined using the DIFF option and the *p* values were corrected by Bonferroni test for pair-wise comparisons. All reported values are least squares means and SEM. Differences were considered significant at *p* < 0.05. Data were analyzed using SAS (Version 9.2, SAS Institute Inc., Cary, NC, USA).

## Results

**Fig. 1:**
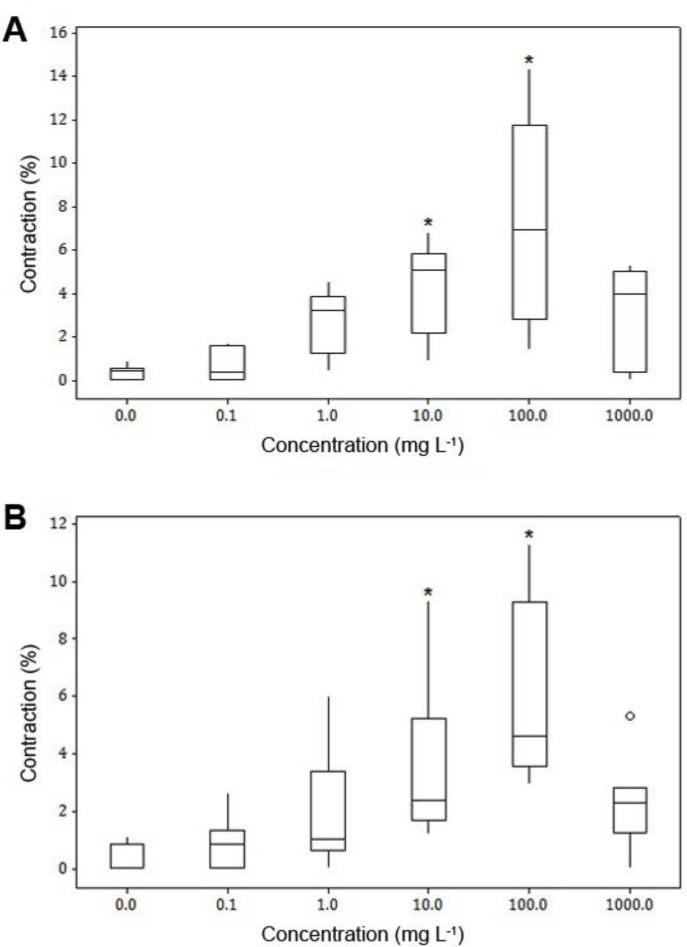
Box plots for effects of ginger extract on basal tonus of healthy sheep reticulum (n = 8) (**A**) and rumen (n = 8) (**B**) preparations with longitudinal orientation. Each box represents the central 50% of the values, the horizontal line within each box represents the median value, and the whiskers indicate the range of values that are within the inner boundary. Values outside the inner fence are plotted as empty circles (ο).

**Organ bath study.** The solvent (distilled water) did not exert an effect on basal tonus of any preparations of sheep reticulum and rumen. Treatment of both tissue strips with 0.1 and 1.0 mg L^-1^ of ginger extract did not show any significant difference with the solvent. The extract increased basal tone of muscles at 10.0 and 100 mg L^-1^ concentrations and showed a difference (*p* < 0.05) with solvent. Addition of the extract at concentration of 1000 mg L^-1^, caused a relaxation in preparations which was contracted with 10.0 and 100 mg L^-1^ concentrations (Figs. 1, A and B).

٭ indicates significant differences compared to control (*p *< 0.05).

**Fig. 2:**
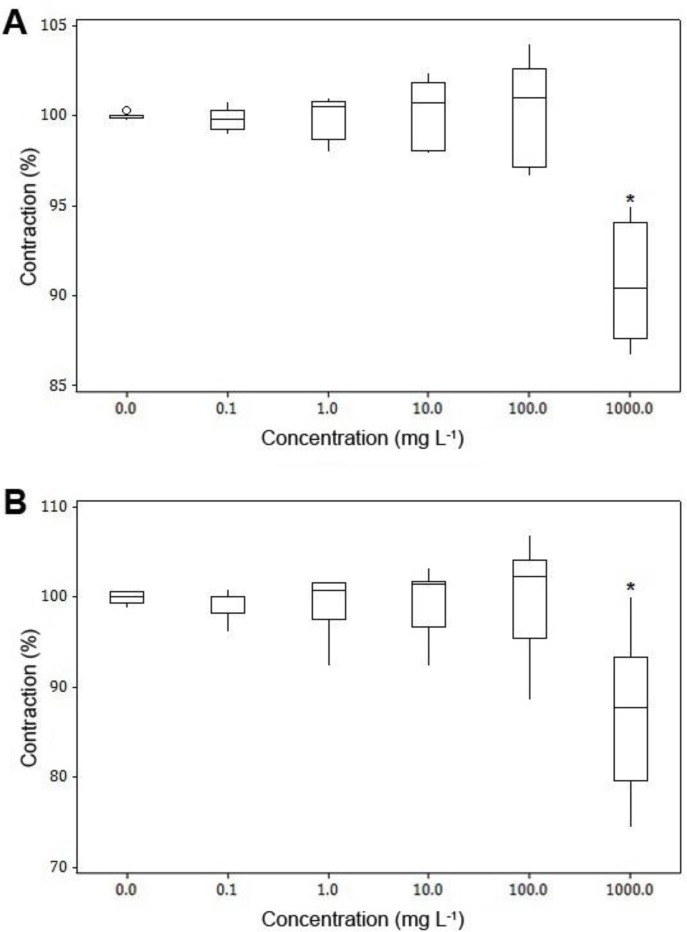
Box plots for effects of ginger extract on ACh-induced contractions in preparations of healthy sheep reticulum (n = 8) (A) and rumen (n = 8) (B) with longitudinal orientation. Each box represents the central 50% of the values, the horizontal line within each box represents the median value, and the whiskers indicate the range of values that are within the inner boundary. Values outside the inner fence are plotted as empty circles (ο).

In muscle strips which were contracted with ACh, treatment of samples with the extract at 0.1 to 100 mg L^-1^ concentrations had no significant effect on contraction of smooth muscles whereas the extract at the concentration of 1000 mg L^-1^, had a relaxant effect on Ach-induced contracted muscles (Figs. 2, A and B).

**Fig. 3:**
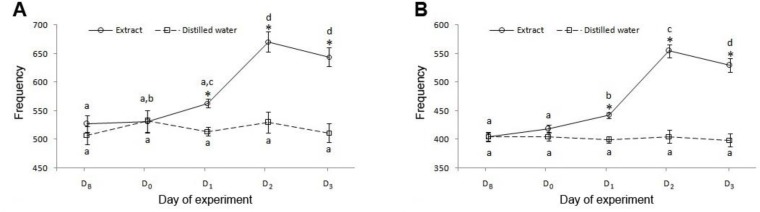
Effects of intraruminal administration of 40 mg kg^-1^ of ginger extract on frequency of contractions of reticulum (**A**) and rumen (**B**) in conscious sheep. Frequency of overall contractions of rumen and reticulum were analyzed for LSM ± SEM in various experiment days.

**Electromyographic study.** The least square means and standard errors for frequency of reticuloruminal contractions in conscious sheep at various experiment days are shown in Table 1 and Fig. 3. The results showed a gradual increase in frequency of overall contractions of reticulum and rumen at the day of administration (D_0_) of ginger extract and the day afterwards (D_1_) in comparison with the day before (D_B_) and with the control group at the same day. Remarkable increase in frequency was seen two days after administration (D_2_) of extract. At the third day (D_3_) after extract administration, the frequency of contractions was yet more than D_0_ and D_1_ but showed a decrease in comparison with D_2_. Statistical analyses were indicative of the significant differences between the frequency of overall ruminal contractions at D_1_, D_2_ and D_3_ compared to D_0_ of the treatment group and the same days of the control group. The frequency of reticular contractions showed significant difference at D_1_, D_2_ and D_3_ of treatment group compared to same days of the control group but the significant increase in frequency of contractions was seen in D_2_ and D_3_ compared to D_0_ at treatment group (*p* < 0.05).

٭ indicates significant differences compared to control (*p *< 0.05).

* indicate significant changes against the control (*p* < 0.05).

^a,b,c,d^ Different letters in each experiment indicate significant difference between days within each group (*p* < 0.05).

**Table 1:** Frequency of overall contractions of rumen (n = 6) and reticulum (n = 4) at the day before administration of extract or distilled water (D_B_), day of intraruminal administrations (D_0_), and one, two and three days after administrations (D_1_, D_2_ and D_3_). Data are presented as least squares means (LSM) and standard error mean (SEM).

	**Treatment**		**Days**						***p*** ** values**		
			**D**_B_	**D**_0_	**D**_1_	**D**_2_	**D**_3_		**Day**	**Treatment**	**Day × Treatment**
**Rumen**	Extract	LSM SE	404.2 7.5	418.5 7.4	441.9 5.3	554.7 11.2	529.7 11.9		< 0.0001	0.0002	< 0.0001
	Distilled water	LSM SE	405.2 7.7	405.2 7.4	399.7 5.3	404.9 11.2	398.9 11.9				
**Reticulum**	Extract	LSM SE	527.5 14.6	531.3 19.3	563.3 7.6	670.5 18.0	644.0 16.3		0.0002	0.0134	0.0003
	Distilled water	LSM SE	507.5 15.7	532.0 19.3	514.0 7.6	530.0 18.0	511.3 16.3				

## Discussion

The role of parasympathetic nervous system in the physiology and pathophysiology of the gastrointestinal motility in different species has been described.^18^ Acetylcholine is the main neurotransmitter in the cholinergic system and causes the contractions of the smooth muscle layers in the forestomachs.^19^ It exerts its effect via activation of muscarinic receptors located directly on smooth muscle cells or nerve cells of the enteric nervous system.^20^ The contractile effects of ACh in reticulum and rumen preparations of healthy sheep have already been demonstrated,^21^^-^^24^ and our results were in agreement with these reports.

Prokinetic action of aqueous-methanolic and aqueous extract of the ginger has been investigated in various contractility models (isolated rat and mouse stomach fundus, rabbit jejunum, rat, mouse and guinea pig ileum and guinea pig colon). The results of the present study showed the probable presence of cholinergic, spasmogenic components (saponins and alkaloids) in the extract which provides its prokinetic action; and the presence of spasmolytic phenolic constituents (6-shogaol, 6-gingerol, 8-gingerol and 10-gingerol) of the calcium antagonist type which contribute in the relaxant action of the ginger. Taken into account all these, it seems convincing that ginger possesses both stimulatory and inhibitory activities.^9^^,^^25^ The results of the *in vitro* study on preparations of sheep reticulum and rumen were both stimulant (at 10.0 and 100 mg L^-1^ concentrations) and relaxant (at the concentration of 1000 mg L^-1^) which might be due to involvement of separate stimulatory or inhibitory pathways in different concentrations of the extract.^25^ The inhibitory effect of the extract on ACh-induced contractions may be due to β-adrenergic stimulatory or cholinergic inhibitory actions of the ginger extract.^26^

It seems two types of receptors are involved in the effect of ginger extract on rumen and reticulum tissues: Low affinity receptors are sensitive to lower concentrations of extract and are responsible for contraction of smooth muscles and high affinity receptors, sensitive to higher concentrations, and capable of inducing a relaxation response in smooth muscles.^24^ This could explain why different concentrations of the extract displayed both stimulant and relaxant effects.

The prokinetic activity of ginger extract through enhancement of small intestinal transit of charcoal meal in mice have been shown in previous studies.^9^^,^^25^ Reversal of the inhibitory effect of cisplatin on gastric emptying in rats by ginger acetone or ethanol extracts or ginger juice has been demonstrated.^27^ Reversed pyrogallol-induced delay in gastric emptying in rodents by oral acetone extract,^28^ and enhanced intestinal transport of a charcoal meal have also been reported.^29^^,^^30^ A bolus of dried ginger rhizome (1.0 g) administered into the gastric lumen of dogs has induced phasic contractions in the antrum.^31^

Hypomotility refers to a reduction in the frequency or strength of contractions, or both.^1^^,^^2^ Reticulorumen hypo-motility or even complete stasis has been associated with hypocalcaemia, endotoxemia, reticuloperitonitis, and acidosis.^2^ The principle of treatment of adult ruminant suspected to have reticulorumen hypomotility is to restore the normal motility in addition to correction of acid base, electrolyte and improvement of the effects of endotoxemia.^1^ A wide variety of drugs have been tested for treatment of forestomach hypomotility in ruminants. Parasympathomimetics such as neostigmine or carbamylcholine should not be used to treat forestomach atony. Neostigmine requires vagal activity to be effective and therefore cannot incite normal primary contractions in atonic rumen and should only be used in hypomotility states. Neostigmine increases the strength of primary contraction without upsetting rhythm or coordination. Carbamylcholine causes hypermotility in sheep although the contractions are uncoordinated, spastic and functionless and thus has no place in the treatment of forestomach dysfunction. Metoclopramide increases the rate of ruminal contractions looking beneficial in rumen hypomotility or motility disturbances arising from vagal nerve damage but its application has been limited to the experimental use.^1^

In the recent years there has been a growing interest on the use of plant extracts and essential oils in treatment of diseases. The studies on the prokinetic effects of ginger and the results of our study provide evidence that ginger having gastrointestinal properties such as a potential to stimulate the reticuloruminal contractions could be used in treatment of reticuloruminal hypomotility in ruminants. Our studies have been done on preparations of healthy animals and in the healthy conscious sheep, hence further studies are needed to investigate the effects of ginger extract on preparations from sheep with reticulorumen hypomotility and in clinically diagnosed hypotonic or atonic conditions.
